# Recombinant production of *Streptococcus equisimilis *streptokinase by *Streptomyces lividans*

**DOI:** 10.1186/1475-2859-6-20

**Published:** 2007-07-05

**Authors:** Elsa Pimienta, Julio C Ayala, Caridad Rodríguez, Astrid Ramos, Lieve Van Mellaert, Carlos Vallín, Jozef Anné

**Affiliations:** 1 Laboratorio de Genética, Departamento de Investigaciones Biomédicas, Centro de Química Farmacéutica. Ciudad de la Habana, Cuba; 2 Laboratory of Bacteriology, Rega Institute, Katholieke Universiteit Leuven, B-3000 Leuven, Belgium

## Abstract

**Background:**

Streptokinase (SK) is a potent plasminogen activator with widespread clinical use as a thrombolytic agent. It is naturally secreted by several strains of beta-haemolytic streptococci. The low yields obtained in SK production, lack of developed gene transfer methodology and the pathogenesis of its natural host have been the principal reasons to search for a recombinant source for this important therapeutic protein. We report here the expression and secretion of SK by the Gram-positive bacterium *Streptomyces lividans*. The structural gene encoding SK was fused to the *Streptomyces venezuelae *CBS762.70 subtilisin inhibitor (*vsi*) signal sequence or to the *Streptomyces lividans *xylanase C (*xlnC*) signal sequence. The native Vsi protein is translocated via the Sec pathway while the native XlnC protein uses the twin-arginine translocation (Tat) pathway.

**Results:**

SK yield in the spent culture medium of *S. lividans *was higher when the Sec-dependent signal peptide mediates the SK translocation. Using a 1.5 L fermentor, the secretory production of the Vsi-SK fusion protein reached up to 15 mg SK/l. SK was partially purified from the culture supernatant by DEAE-Sephacel chromatography. A 44-kDa degradation product co-eluted with the 47-kDa mature SK. The first amino acid residues of the *S. lividans*-produced SK were identical with those of the expected N-terminal sequence. The Vsi signal peptide was thus correctly cleaved off and the N-terminus of mature Vsi-SK fusion protein released by *S. lividans *remained intact. This result also implicates that the processing of the recombinant SK secreted by *Streptomyces *probably occurred at its C-terminal end, as in its native host *Streptococcus equisimilis*. The specific activity of the partially purified *Streptomyces*-derived SK was determined at 2661 IU/mg protein.

**Conclusion:**

Heterologous expression of *Streptococcus equisimilis *ATCC9542 *skc-2 *in *Streptomyces lividans *was successfully achieved. SK can be translocated via both the Sec and the Tat pathway in *S. lividans*, but yield was about 30 times higher when the SK was fused to the Sec-dependent Vsi signal peptide compared to the fusion with the Tat-dependent signal peptide of *S. lividans *xylanase C. Small-scale fermentation led to a fourfold improvement of secretory SK yield in *S. lividans *compared to lab-scale conditions. The partially purified SK showed biological activity. *Streptomyces lividans *was shown to be a valuable host for the production of a world-wide important, biopharmaceutical product in a bio-active form.

## Background

Streptokinases are proteins translocated to the growth medium by many strains of beta-haemolytic streptococci. Streptokinase is not an enzyme *per se *but rather a potent activator that interacts with plasminogen to form a stoichiometric 1:1 complex. This interaction results in the activation of plasminogen to plasmin, which is the active fibrinolytic component of the circulatory system [1]. SK was the first drug introduced as a therapy for acute myocardial infarction more than 40 years ago [2]. It is now the leading fibrinolytic agent in the treatment of thromboembolic conditions [3] and is included in the World Health Organization Model List of Essential Medicines.

The *Streptococcus equisimilis *H46A *skc *gene encoding streptokinase has been cloned and expressed in several heterologous hosts due to the pathogenicity of its natural host. Haemolytic streptococci secrete several toxins that complicate the downstream purification. Besides, genetic modification of the natural host is restricted as rather few genetic tools are available. As a result, the recombinant production of this protein in *E. coli *has been widely used, including the use of the protein SKC-2 naturally secreted by *Streptococcus equisimilis *ATCC 9542 [4,5]. High-level expression of *skc *in *E. coli *has been reported, but the formation of inclusion bodies consisting of highly aggregated SK molecules makes its recovery in an active form difficult [4,5]. High level of intracellular SK has also been obtained during continuous fermentation of recombinant *Pichia pastoris *but protein recovery requires cell lysis [6].

Since the recovery of extracellular proteins is generally easier than that of cytoplasmic proteins, the expression and subsequent secretion of SK have been studied in several heterologous hosts like *Escherichia coli*, *Bacillus subtilis *and *Pichia pastoris *[7-9]. In case of *B. subtilis*, the use of the six-extracellular-protease-deficient strain, WB600, greatly improved the yield of recombinant SK. The protein was also secreted into the culture medium by *P. pastoris*, but it was found to be heavily glycosylated. The biological activity of both secreted streptokinases was proved. A recent study using *Schizosaccharomyces pombe *as host, reported the expression of SK and its secretion into the periplasmic fraction without glycosylation and significant degradation or modification. However, conventional chromatographic approaches used before to purify SK from other hosts were inadequate because of cofractionation of a few proteins of similar size with SK through all the chromatographic steps [10].

As it is not possible to predict which host will be the best for the production of a protein, the aim of this work was to evaluate *Streptomyces lividans *as host for recombinant production of SK. *S. lividans *has been successfully used for the production of several proteins of bacterial and eukaryotic origin [11-13]. The advantages of the *S. lividans *host include its natural ability to secrete high levels of bioactive molecules into the extracellular medium, limited protease activity, its biological safety and well-established fermentation technology [14]. In the present study, the *S. lividans *system has been tested for the secretory production of the streptokinase from *Streptococcus equisimilis *group C by using the Sec and the recently described twin-arginine translocation (Tat) pathway in *S. lividans *[15]. The sequence encoding mature SK was fused to the Sec-dependent signal sequence of *Streptomyces venezuelae *CBS762.70 subtilisin inhibitor [16] and the twin-arginine signal sequence of *S. lividans *xylanase C [17], respectively. SK production in *S. lividans *was evaluated and purification of the secreted SK protein was carried out.

## Results

### Construction of SK expression/secretion vectors

In order to establish the expression and secretion of SK from *Streptococcus equisimilis *ATCC9542 in *S. lividans*, the *skc-2 *gene was amplified by PCR using chromosomal DNA as template and finally cloned in appropriate vectors in *Streptomyces*. The constructed expression/secretion vectors pOVsiSK and pOXlnCSK encode the fusion proteins Vsi-SK and XlnC-SK, respectively (Table 1). Vsi-SK consists of the Sec-dependent Vsi signal peptide, the first two amino acid residues of mature Vsi followed by the mature SK, while XlnC-SK is composed of the Tat-dependent XlnC signal peptide, the first three amino acids of mature XlnC and mature SK. In this way, the signal peptidase cleavage sites of Vsi and XlnC are remained and a proper processing of the precursor proteins is achieved. Both fusion genes were placed under control of the *vsi *promoter, of which has been proved that it efficiently promotes heterologous gene expression [13,16].

**Table 1:** Plasmids used in this study.

**Name**	**Relevant properties**	**Source or reference**
pGEM-SK	pGEM^®^-T Easy derivative containing the *Streptococcus equisimilis skc-2 *gene	This work
pBS-CBSS	pBluescript KS(+) derivative containing the *Streptomyces venezuelae vsi *promoter and part of the mature *vsi *gene	[16]
pBSVX	pBluescript KS(+) derivative containing the *Streptomcyes venezuelae vsi *promoter and the signal sequence of *Streptomyces lividans xlnC*	[17]
pBSVXM	pBSVX derivative containing a unique *Eco*RI site downstream of the signal sequence of *Streptomyces lividans xlnC*	This work
pUWL-218	*Escherichia coli*-*Streptomyces *shuttle vector, multiple cloning site, Ap^R^, Tsr^R^	[40]
pOW15	pUWL-218 derivative *E. coli-Streptomyces *shuttle vector containing the *oriT *fragment for interspecies DNA conjugation.	Rosabal et al., unpublished.
pOVsiSK	pOW15 derivative containing the *Streptomyces venezuelae vsi *promoter, and signal sequence, and the *Streptococcus equisimilis skc-2 *gene	This work
pOXlnCSK	pOW15 derivative containing the *Streptomyces venezuelae vsi *promoter, the *Streptomyces lividans xlnC *signal sequence and the *Streptococcus equisimilis skc-2 *gene	This work

### Secretion of SK by *S. lividans*

**Figure 1:**
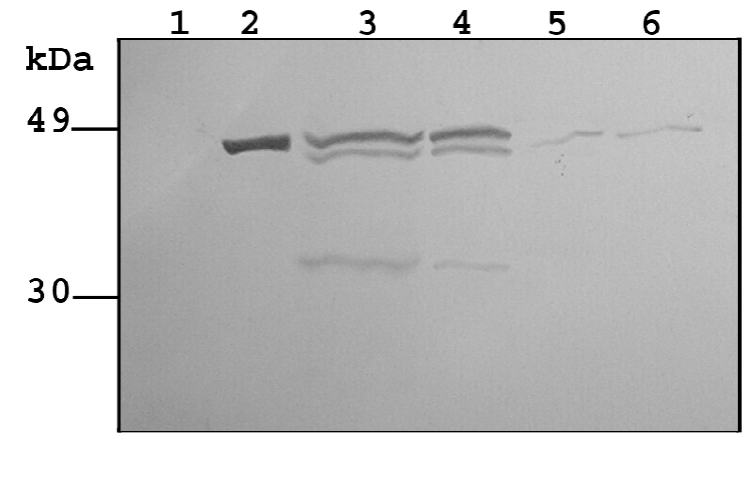
**Immunodetection of SK in extracellular fractions**. Proteins from culture supernatants were precipitation with a mixture of chloroform and methanol (1:3, v/v). In each lane, proteins according to 100 μl spent culture medium were loaded. *Lane 1, S. lividans *[pOW15] 40 h; *lane 2*, 60 ng SK standard; *lane 3*, *S. lividans *[pOVsiSK] 30 h; *lane 4*, *S. lividans *[pOVsiSK] 40 h; *lane 5*, *S. lividans *[pOXlnCSK] 30 h; *lane 6*, *S. lividans *[pOXlnCSK] 40 h.

*S. lividans *transformants carrying pOVsiSK and pOXlnCSK, respectively, were grown at lab scale in rich BTSB medium and at several time intervals the presence of SK in the culture filtrate was assessed by Western blot analysis. A clear SK-specific band of about 47 kDa and smaller immunoreactive bands of approximately 44 kDa and 32 kDa could be observed in culture supernatant of *S. lividans *[pOVsiSK] at 30 and 40 h of growth (Fig. 1, lanes 3 and 4). SK was faintly detectable in culture filtrate of *S. lividans *[pOXlnCSK] upon 30 and 40 h growth (Fig. 1, lanes 5 and 6). No SK-specific immunoreactive proteins could be detected in cell lysates of *S. lividans *carrying pOVsiSK or pOXlnCSK (data not shown), which indicated that the produced preproteins did not accumulate inside the cell and were efficiently translocated through the cell membrane.

**Figure 2:**
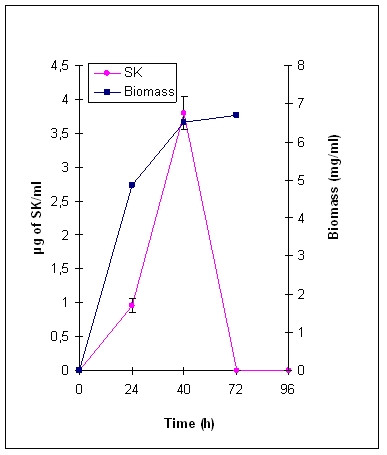
**Secretory yield of recombinant SK correlated with biomass of *Streptomyces lividans *[pOVsiSK] grown in lab-scale conditions**. SK concentration was determined by means of ELISA. Growth was estimated by measuring biomass dry weight (mg/ml), standard errors were between 0.1 and 0.2.

The amount of SK secreted by the *S. lividans *transformants, measured by means of ELISA, reached a maximum around 40 h and then decreased. Upon 40 h growth, up to 4 mg SK/l medium was measured in culture supernatant of *S. lividans *[pOVsiSK] (Fig. 2), while 100–150 μg SK/l was detected in the extracellular fraction of 40-h *S. lividans *[pOXlnCSK] cultures. These results indicate that SK can be translocated via both the Sec and the Tat pathway in *S. lividans*, but the Sec-routed secretion leads to higher levels of the recombinant protein.

In consequence of the poor SK yield using the XlnC signal peptide as mediator for translocation, only *S. lividans *[pOVsiSK] was tested under fermentation conditions. Using small-scale fermentation conditions, the secretory SK production reached up to 15 mg SK/l, which corresponds to a fourfold improvement of secretory SK yield in *S. lividans *compared to lab-scale conditions.

### Purification of recombinant SK secreted by *S. lividans*

**Figure 3:**
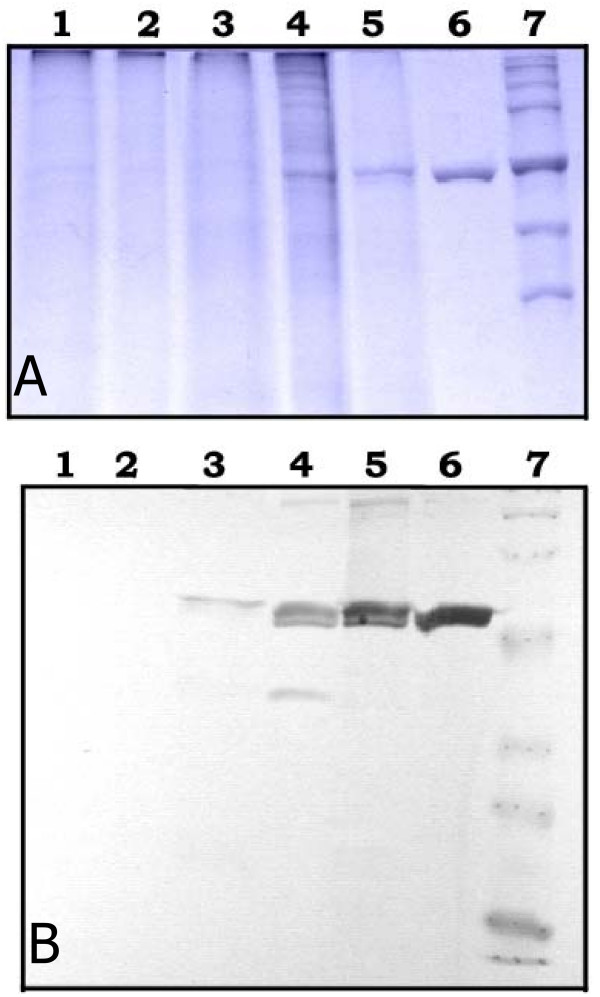
**Purification of extracellular SK from *S. lividans *culture supernatants upon small-scale fermentation**. (A) 10% SDS-PAGE stained with Coomassie blue R-250, and (B) Immunoblotting analysis using a monoclonal anti-SK antibody. *Lane 1*, 25 μg of crude extract of *S. lividans *TK24 [pOW15]; *lane 2*, 25 μg of material precipitated with (NH_4_)_2_SO_4 _of *S. lividans *TK24 [pOW15]; *lane 3*, 25 μg of crude extract of *S. lividans *TK24 [pOVsiSK]; *lane 4*, 25 μg of proteins precipitated with (NH_4_)_2_SO_4 _of *S. lividans *TK24 [pOVsiSK]; *lane 5*, 25 μg of pooled anion exchange chromatography protein fractions with 58% purity; *lane 6*, 1 μg of SK standard; *lane 7*, Broad-range protein molecular weight markers.

Having defined the fermentation conditions for the secretory production of recombinant SK, the protein was purified from the extracellular culture fraction. The protein fraction obtained through ammonium sulfate precipitation (45% saturation) was dissolved in 20 mM Tris-HCl (pH 6.0), dialyzed against the same buffer and then applied on a DEAE-Sephacel column. The proteins were eluted in 20 mM Tris-HCl, 150 mM NaCl, pH 6.0. Samples from the various purification steps were analysed by SDS-PAGE followed by Coomassie staining (Fig. 3A) and immunodetection of the recombinant SK using a monoclonal anti-SK antibody (Fig. 3B). Samples from culture supernatant and 45% ammonium sulphate saturation fraction of *S. lividans *TK 24 [pOW15] were included as negative controls (Fig. 3, lanes 1 and 2). This experiment revealed that the purified proteins correspond to SK. Pooled elution fractions with a purity grade of 58% contained the 44-kDa degradation product which co-eluted with the 47-kDa mature SK.

In order to determine the specificity of signal peptidase processing and the nature of the approximately 44-kDa protein, an N-terminal sequence was carried out on both the 47- and 44-kDa proteins obtained from *S. lividans *TK 24 [pOVsiSK] culture supernatant. N-terminal residues of both purified proteins were those predicted from the sequence (Table 2). The Vsi signal peptide was thus correctly cleaved off and the N-terminus of mature Vsi-SK released by *S. lividans *remained intact. This result also implicates that the processing of mature Vsi-SK secreted by *Streptomyces *occurred at its C-terminal end.

**Table 2:** Amino acid sequence of the fusion region of preVsi-SK and the N-terminal amino acid sequence of the 47- and 44-kDa proteins obtained from *S. lividans *TK 24 [pOVsiSK] culture supernatant.

**Protein**	**Amino acid sequence**
PreVsi-SK (fusion region)	...A Q A ↓ E A *I A G P E W L L*...
N-terminus 47-kDa rSK	E A *I A G P E W L L*...
N-terminus 44-kDa rSK	E A *I A G P E W L L*...

↓: Signal peptidase cleavage site, SK sequence in italics

The specific activity of the partially purified proteins secreted by *S. lividans *[pOVsiSK] was amounted to 2661 IU/mg protein (Table 3). In consequence of incompatibility between the crude culture medium and the chromogenic substrate assay, we were not able to determine the initial specific activity of SK.

**Table 3:** Secretory production of recombinant SK by *S. lividans *TK 24 [pOVsiSK].

**Sample**	**Volume (ml)**	**SK activity (IU/ml)**	**Protein concentration (mg/ml)**	**ELISA SK (mg/ml)**	**Specific activity (IU/mg)**
Culture supernatant	1000	ND	1.04	0.015	ND
DEAE eluates with 58% purity	16	444 ± 24	0.17	0.021	2661 ± 29

ND: No determined.

## Discussion

In the present study, it was shown that SK from *Streptococcus equisimilis *ATCC9542 could be efficiently secreted in a bio-active form via the Sec pathway in *Streptomyces lividans*. Sec-routed secretion was obtained by using the regulatory signal sequences of *S. venezuelae *CBS762.70 subtilisin inhibitor gene. The Tat translocation route was also tested for the secretion of SK in *S. lividans *by means of a fusion of SK to the Tat-dependent signal peptide of *S. lividans *xylanase C. Yield was about 30 times higher when the SK was fused to the Sec-dependent Vsi signal peptide compared to the fusion with the Tat-dependent XlnC signal peptide. Although the use of the Tat pathway in most cases does not result in higher production yield compared to Sec-mediated secretion (e.g. Schaerlaekens et al. 2004), some proteins need to be secreted via the Tat pathway to obtain their bio-active conformation. This is the case for the homologous protein xylanase C [18], but also for the heterologous enhanced green fluorescent protein (EGFP) [19].

The maximum level of SK secreted by *S. lividans *was 15 mg/l of culture, but as a result of incompatibility between the crude culture medium and the chromogenic substrate assay, we were not able to determine the initial activity of *Streptomyces*-derived SK. SK secreted by recombinant *S. lividans *was partially purified (58% purity) and was found biologically active with an specific activity of 2661 IU/mg protein. In addition, it is not possible to compare reliably the plasminogen activity of the partially purified SK secreted by *Streptomyces *with the initial SK activity secreted by other hosts like: *Streptococcus equisimilis *(100–150 IU/ml)*, E. coli *(1000–1500 IU/ml), *P. pastoris *(3200 IU/m1) or *S. pombe *(2450 IU/m1) [9,10]. However, it is possible to establish a relative comparison with the total yield (24.5 mg/l) and the initial specific activity of SK secreted by *S. pombe *(1581 IU/mg protein) [10].

SK has a tendency to degrade very easily [20,21]. Several hosts, including the native host *Streptococcus equisimilis*, produce at least two major forms of SK [7,8,22]: the intact mature SK with a molecular mass of 47 kDa and a 44-kDa degradation product. This degradation product lacks 31 or 32 C-terminal residues whereas it retains the plasminogen activation capability [23]. Furthermore, C-terminal deletion mutants of SK lacking 40 [24] or 41 amino acids [25] exhibited normal plasminogen activator function. In addition to the 47- and 44-kDa bands, a 32-kDa degradation product was detected by Western blot. Since SK proteins which lack 18 or more N-terminal or 51 or more C-terminal amino acid residues are unlikely to be effective thrombolytic agents [24], the 32-kDa SK-related protein missing about 135 aa residues was not further investigated.

It was demonstrated that the post-translational modification at the C-terminus of native SK was caused by chymotrypsin-like activity [23]. Similar degradation of recombinant SK has been also reported to occur in heterologous hosts such as *Streptococcus sanguis *[23] and *E. coli *[26]. Chymotrypsin-like activity and several genes encoding chymotrypsin-like serine proteases have been reported in *S. lividans *66 [27,28]. Since the first amino acid residues of the *S. lividans*-produced Vsi-SK were identical to those of the expected N-terminal sequence, the recombinant protein was proteolytically degraded at its C-terminal end.

In case of recombinant SK production in *Lactococcus lactis*, the protease susceptibility and hence the productivity of SK was dependent on the pH of the culture and the initial phosphate concentration of the medium. Suppression of the acid tolerance response, by which protease expression is induced, enhanced the SK yield 2.5 fold [29]. Results of a differential scanning calorimetry study on *E. coli*-derived recombinant *S. equisimilis *SK firmly indicated that at neutral and basic pH, the recombinant SK from *Streptococcus equisimilis *group C (ATCC 9542) has four domains, whereas gentle changes in the experimental conditions, such as mild acidification or increase in the NaCl concentration, decreased this number [30]. Consequently, pH and ionic strength of the production medium define the conformational status of SK and are thus important factors determining the protease susceptibility of the recombinant protein.

The specific activity of the partially purified SK (58% purity) secreted by *S. lividans *[pOVsiSK] was determined at 2661 IU/mg protein. We believe that further up-scaling of the fermentation process and optimisation of production medium and purification protocol, will surely improve yield of recombinant bio-active SK in *S. lividans*.

## Conclusion

Heterologous expression of *Streptococcus equisimilis *ATCC9542 *skc-2 *in *Streptomyces lividans *was successfully achieved. SK can be translocated via both the Sec and the Tat pathway in *S. lividans*, but yield was about 30 times higher when the SK was fused to the Sec-dependent Vsi signal peptide compared to the fusion with the Tat-dependent signal peptide of *S. lividans *xylanase C. Small-scale fermentation led to a fourfold improvement of secretory SK yield in *S. lividans *compared to lab-scale conditions. The plasminogen activity of the partially purified SK (58% purity) secreted by *S. lividans *[pOVsiSK] was determined at 2661 IU/mg protein. Once more, *Streptomyces lividans *was shown to be a valuable host for the production of a world-wide important, biopharmaceutical product in a bio-active form.

## Methods

### Bacterial strains and growth conditions

*E. coli *TG1 was used as host for cloning purposes. Culture conditions for *E. coli *were as described by Sambrook et al. [31]. *Streptococcus equisimilis *ATCC9542 cells were grown as described by Estrada et al. [4]. *Streptomyces lividans *TK24 was selected as host for heterologous protein production. Protoplast formation and subsequent transformation of *S. lividans *were carried out as described by Kieser et al. [32]. Regeneration of *S. lividans *protoplasts and selection of transformants was carried out on MRYE medium [33]. When appropriate, thiostrepton (50 μg/ml in solid medium or 10 μg/ml in liquid medium) was added. Spore suspensions of *S. lividans *TK24 and derivatives were stored at -70°C in 20% (v/v) glycerol. Primary cultures of *S. lividans *strains were routinely cultured for 72 h (28°C, 240 rpm) in BTSB, which is a modified version of the medium described by Dyson and Schrempf [34]: 10% sucrose, 1% yeast extract, 1% glucose, 0.5% NaCl, 0.5% soya flour, 1.7% tryptone, 0.25% K_2_HPO_4_, pH 7.2. For monitoring recombinant protein expression and secretion, 1-ml primary cultures were inoculated to 0.5-L shake flask containing 0.1 L of BTSB medium and grown for 40 h at 28°C and 300 rpm. For production of SK, the recombinant strain was cultured for 48 h (28°C, 350 rpm) in a 2.5-L MBR reactor containing 1.5 L BTSB medium. The pH was controlled at 7.0 by the addition of 5 N NaOH.

### Plasmid construction and recombinant DNA technology

DNA manipulations were carried out following standard procedures [31,32]. Restriction endonucleases and DNA-modifying enzymes were from Invitrogen and Roche Diagnostics. Upon isolation of *S. equisimilis *ATCC9542 chromosomal DNA as described by Estrada et al. [4], the *skc-2 *gene was PCR-amplified from the chromosome under standard conditions using the oligonucleotides SK-F1 and SK-R1 (see Table 4) and *Taq *polymerase. To allow in-frame fusion of the *skc-2 *gene downstream the signal sequences, the oligonucleotide SK-F1 was designed with an *Eco*RV site (GATATC) in its 5'end. Consequently, a silent mutation was introduced in the first codon of the mature part of the *skc-2 *gene: ATT was replaced by ATC, both encoding isoleucine.

**Table 4:** Oligonucleotides used in this study.

**Name**	**Sequence (5'-3' direction)**	**Restriction sites (in italic)**
SK-F1	*GATATC*GCTGGACCTGAGTGGCTG	*Eco*RV
SK-R1	*AGATCT*TTATTTGTCGTTAGGGTTATCAG	*Bgl*II
PBSXylE-F	CCGGGCTGCAGGAA**G**TCGATTCGGGAGCG	-
PBSXylE-R	CGCTCCCGAATCGA**C**TTCCTGCAGCCCGG	-

The mutagenic base is in bold face.

The 1245-bp PCR fragment was ligated into pGEM^®^-T Easy (Promega) and the resulting plasmid was denominated pGEM-SK. The DNA sequence was verified using the Thermo Sequenase Primer Cycle Sequencing Kit with 7-deaza-dGTP on an ALFexpress apparatus (Amersham Biosciences, Rainham, UK). Subsequently, the 1.3-kb *Eco*RV/*Eco*RI fragment of pGEM-SK was cloned into pBS-CBSS [16] successively treated with *Dra*II, Klenow polymerase and *Eco*RI. The unique *Dra*II site in pBS-CBSS is located two codons downstream the signal peptidase recognition site. *skc-2 *was also cloned into pBSVXM, a derivative of plasmid pBSVX [17] missing an *Eco*RI site. To remove the *Eco*RI site located upstream the *vsi *promoter in pBSVX, a site-directed mutagenesis was carried out by means of PCR using *Pfu *polymerase and the mutagenic oligonucleotides PBSXylE-F and -R (Table 4), which contain the desired mutation. As such, a unique *Eco*RI site located downstream the *S. lividans xlnC *signal sequence was available. In order to insert the *skc-2 *gene fused to the third codon of mature *xlnC*, the vector pBSVXM was digested with *Nsi*I, treated with T4 DNA polymerase removing the 3'-protruding ends and finally treated with *Eco*RI. DNA sequence analyses of the newly constructed fusion genes confirmed their correctness.

Finally, both expression/secretion cassettes were isolated as *Bam*HI/*Eco*RI-fragments and ligated in *Bam*HI/*Eco*RI-digested pOW15. The vector with the *vsi *signal sequence was designated pOVsiSK and the vector with the *xlnC *signal sequence was denominated pOXlnCSK. Plasmids used in this study are listed in Table 1.

### Detection of SK

The detection of SK in culture supernatants and cell lysates of *S. lividans *transformed with pOVsiSK or pOXlnCSK was performed using Western Blot and immunodetection. Gel electrophoresis of proteins was carried out on 10% SDS-polyacrylamide gels [35]. Separated proteins were visualized by Coomassie brilliant blue staining or transferred to a Hybond™-C extra membrane (GE Healthcare) by using a semidry transfer cell (Biometra) according to the manufacturer's recommendations. SK was detected using a mouse anti-SK monoclonal antibody (produced by Center for Genetic Engineering and Biotechnology, Sancti Spiritus, Cuba). HRP-conjugated goat anti-mouse antibody (Promega) was used as secondary antibody. Immunoreactive bands were visualized by brief exposure to 3,3-diaminobenzidine or 4-chloro naftol (Sigma). Cell lysates were obtained according to Pimienta et al. [36]. The protein content of culture supernatants, cell lysates and purified fractions was determined using the Bradford method [37].

The molecular size and the purification degree of recombinant SK protein were estimated from densitometric scanning of Coomassie brilliant blue-stained gels using a GENE GENIUS gel documentation system and GeneTools software (Syngene).

SK present in culture supernatants or anion exchange chromatography eluates was quantified by means of a general sandwich ELISA protocol (Abrahantes et al. unpublished results). The SK standard was kindly supplied by the Development Division, Center for Genetic Engineering and Biotechnology, Havana, Cuba. The coefficient of variation of the ELISA tests was less than 10%.

SK activity was monitored spectrophotometrically at 405 nm in a coupled SK-plasminogen assay employing the chromogenic substrate S-2251 (Kabi, Sweden) according to Hernández et al. [38]. The specific activity (IU/mg) was calculated by dividing the SK activity (IU/ml) with protein concentration (mg/ml).

### Protein purification and chromatography

For purification of SK from recombinant *S. lividans *cultures, strains were grown for 2 days in 200 ml BTSB medium. Then, cultures were centrifuged and the mycelium was resuspended in 0.1 L of water. This suspension was transferred to 1.5 L BTSB in the MBR reactor and bacterial growth was continued for 2 days. Culture supernatant proteins were precipitated by addition of (NH_4_)_2_SO_4 _(45% saturation, 4°C) and collected by centrifugation (Hettich Universal 32R centrifuge, Sorvall, 1620A rotor, 4°C, 20 min, 8000 × g). The protein pellets were left overnight at 4°C in 0.1 L of 20 mM Tris-HCl buffer, pH 6.0. Then, the protein solution was dialyzed against 20 mM Tris-HCl buffer (pH 6.0) at 4°C for 20 h and was finally applied on a DEAE Sephacel column equilibrated with 20 mM Tris-HCl buffer, pH 6.0. The column was extensively washed with the mentioned buffer followed by 1 column volume of 20 mM Tris-HCl, 20 mM NaCl, pH 6.0. SK protein elution from the DEAE Sephacel column was carried out with 3 column volumes of 20 mM Tris-HCl, 150 mM NaCl, pH 6.0 at a flow rate of 0.5 ml/min. One ml fractions were collected. Fractions containing SK with a similar degree of purity, determined by means of SDS-PAGE followed by Coomassie staining, were pooled.

### N-terminal amino acid sequence analysis

The purified SK was subjected to SDS-PAGE and blotted on a Hybond-P membrane (GE healthcare) as described by Ausubel et al. [39]. After Coomassie staining, the relevant protein bands were excised and subjected to sequencing. The N-terminal amino acid sequence of recombinant SK was determined by Edman degradation using an automatic 477A-1201 protein sequencing system (Applied Biosystems).

## Competing interests

The author(s) declare that they have no competing interests.

## Authors' contributions

EP participated in the design of the study, performed clonings, strain constructions and fermentation, analyzed the data and wrote the manuscript. JCA participated in design and performance of protein purification. CR participated in design and performance of protein purification. AR participated in design and performance of growth conditions. LVM, CV and JA participated in design of the study and commented the manuscript. All authors have read and approved the manuscript.
